# Efficient Adsorption of Lead on Hydro-Pyrochar Synthesized by Two-Step Conversion of Corn Cob in Magnesium Chloride Medium

**DOI:** 10.3390/toxics13060459

**Published:** 2025-05-30

**Authors:** Marija Simić, Jelena Petrović, Marija Koprivica, Marija Ercegović, Jelena Dimitrijević, Nikola S. Vuković, Núria Fiol

**Affiliations:** 1 Institute for Technology of Nuclear and Other Mineral Raw Materials, 11000 Belgrade, Serbia; j.petrovic@itnms.ac.rs (J.P.); m.koprivica@itnms.ac.rs (M.K.); m.ercegovic@itnms.ac.rs (M.E.); j.dimitrijevic@itnms.ac.rs (J.D.); n.vukovic@itnms.ac.rs (N.S.V.); 2 LEPAMAP-PRODIS Research Group, Universitat de Girona, 17004 Girona, Spain; nuria.fiol@udg.edu

**Keywords:** hydrothermal carbonization, pyrolysis, hydro-pyrochar, Pb adsorption, reusability, corn cob

## Abstract

This study used widely available waste biomass, corn cob (CC), to remove Pb ions from aqueous solutions. A two-step conversion of this material was carried out to improve its adsorption characteristics. Firstly, CC was prepared by hydrothermal carbonization; afterward, the obtained hydrochar was doped by MgCl_2_ and pyrolyzed. The synthesized hydro-pyrochar (HCC-Mg) was used for adsorption experiments in a batch system. The surface and structural properties of HCC-Mg were characterized by SEM-EDX and FTIR analysis before and after Pb adsorption. Kinetic and isotherm models were applied to the experimental results. It was confirmed that Pb adsorption on HCC-Mg occurred rapidly, with a maximum adsorption capacity of 87.08 mg/g. The pseudo-second-order model best described the adsorption process, while the best fit of the experimental data was observed with the Sips isotherm model. The results of this study showed that the capacity of the synthesized HCC-Mg material had increased more than 14 times compared with raw CC. In addition, the synthesized material had the potential to be reused for at least five cycles with minimal loss of adsorption capacity and efficiency. Moreover, the results confirmed that HCC-Mg can be used as an efficient, sustainable adsorbent of Pb from polluted water.

## 1. Introduction

Environmental pollution caused by heavy metals presents significant risks to both human health and ecosystems. Lead exposure is linked to long-term neurological damage, especially in children, affecting cognitive development and behavior. Lead accumulates in the body, causing harm to bones, the nervous system, and kidney function. Additionally, lead adversely impacts aquatic ecosystems and plant growth [1]. Mitigation strategies include reducing industrial emissions, banning lead-based products, and enforcing strict regulations to limit exposure and environmental contamination [2].

Adsorption is a promising method for toxic-pollutant removal due to its high efficiency, cost-effectiveness, and environmental sustainability. Different materials, such as activated carbon, biochars, and zeolites, have been investigated as adsorbents and have shown significant potential in heavy-metal-ion removal from water solutions [3,4,5,6]. This technique has proven suitable, selective, and effective for pollutant removal, preventing harmful environmental accumulation [7]. Recently, advanced porous materials such as metal–organic frameworks (MOFs) and covalent organic frameworks (COFs) have also shown great potential for metal ion removal due to their tunable structures and high surface areas [8,9].

Several studies have highlighted that waste biomass can be converted into carbon-rich materials through the hydrothermal carbonization (HTC) process and pyrolysis, effectively removing heavy metals [3,10]. In their research, Su et al. (2023) showed that Cd ions could be effectively removed by hydrochar from kitchen waste [11]. Although hydrochars are effective adsorbents for removing metal ions from wastewater, their adsorption efficiency is limited by a relatively low specific surface area and a lack of active functional groups available for interaction. Many studies have shown that modifying the hydrochar surface can enhance the quantity of active functional groups and improve adsorption capacity [3,12]. This way, Petrović et al. modified grape pomace hydrochar with KOH [13]. This modification increased surface porosity and the availability of active functional groups and improved the material’s adsorption characteristics. Biochars derived from numerous waste materials have also been used as adsorbents, and some of them have exhibited excellent adsorption performance [10]. Biochars derived from corn stalk and rice husk were investigated as adsorbents of Pb^2+^ ions [14]. Deng et al. investigated the adsorption behavior and mechanisms of Cd and Ni on rice straw biochars in single and binary metal systems. They showed that pyrolysis temperature affected the adsorption behavior [15]. To provide more active adsorption sites for interaction with Cd and its removal from aqueous solutions, modified biochar derived from rice husk was investigated. The results of this study show that the capacity of modified rice husk increased nearly 14 times compared with the raw material [16].

Corn cob, a widely available agricultural waste material, has been studied as an adsorbent for heavy metals due to its high cellulose content and porous structure. Scientific studies have shown that corn cob can be utilized as a low-cost and sustainable material for metal removal, although its adsorption capacity is relatively low [17,18]. Based on our previous investigations, it has been established that this native waste material demonstrates inadequate adsorption properties concerning the removal of heavy metals [19,20]. Further modifications, such as thermochemical conversion and chemical activation, can enhance its adsorption capacity, making it a promising material for heavy metal removal [21]. Hence, it is essential to find a simple and effective method for modification of this material.

In this study, the ability of CC hydro-pyrochar, a novel material obtained through hydrothermal carbonization (HTC) and further modified by pyrolysis with MgCl_2_, to act as an adsorbent for Pb ion removal from aqueous solutions, was systematically investigated for the first time. While there have been numerous studies on the adsorption of heavy metals on natural sorbents and their hydro- and biochars, our study presents a unique approach. The material in our research was primarily obtained through HTC treatment of CC, making it a hydrochar-based material. The pyrolysis process was used as a modification step, aimed at introducing new functional groups from magnesium salts onto the surface of the hydrochar. To the best of our knowledge, this material has not been previously applied for the removal of lead (Pb) from aqueous solutions. Thus, our work introduces a novel material that combines hydrothermal carbonization with post-pyrolysis modification for heavy metal removal. Adsorption experiments were performed in a batch system, and detailed characterization was done. Specifically, the study aimed to (1) synthesize a new material with improved adsorption characteristics; (2) characterize the synthesized hydro-pyrochar; (3) investigate the novel adsorbent for Pb ion removal from aqueous solutions; and (4) study the adsorption mechanism of Pb (II) onto HCC-Mg.

## 2. Materials and Methods

### 2.1. Material Synthesis

The CC was collected from a local cornfield in Belgrade, Serbia, ground, air-dried, and used for hydro-pyrochar synthesis. Approximately 10 g of CC was mixed with 150 mL of water and carbonized in a laboratory autoclave (Carl Roth, Model II) at 220 °C. After the reaction, the suspension was filtered, rinsed with distilled water, dried to constant weight, and the obtained hydrochar (HCC) was impregnated with MgO, according to Petrović et al., 2023 [13]. About 1 g of HCC was mixed with 20 mL of distilled water and stirred for 30 min. Afterward, 2 M MgCl_2_·6H_2_O was added to the suspension, the pH was adjusted to 10 by adding 5 M NaOH, and the mixture was stirred for 4 h at 60 °C. The prepared material was further pyrolyzed for 1 h in a quartz crucible in a pyrolysis oven at 300 °C, with a heating rate of 10 °C/min, under an inert N_2_ atmosphere with a 50 mL/min flow rate. After pyrolysis, the material was filtered, rinsed with distilled water, dried in an oven at 105 °C to a constant weight (HCC-Mg), and used for further experiments.

### 2.2. Metal Solution Preparation

The primary Pb(II) standard solution was prepared by dissolving a desired amount of Pb(NO_3_)·3H_2_O in distilled water. All used chemicals were of p.a. purity grade.

### 2.3. Material Characterization

The surface morphology of the HCC-Mg before and after Pb adsorption was examined by scanning electron microscopy and energy dispersive X-ray analysis (SEM-EDX). SEM-EDX analysis was carried out using a JEOL JSM-700 1F (JEOL (GERMANY) GmbH, Freising, Germany).

Fourier-transform infrared spectroscopy (FTIR), carried out using a Thermo Nicolet 6700 FTIR, was used to analyze the chemical structure of HCC-Mg before and after Pb adsorption.

The determination of the pH_PZC_ of HCC-Mg in the KNO_3_ solution (0.001 mol/L) was described in our previous study [20,21].

### 2.4. Adsorption Experiments

All experiments were performed in a batch system at room temperature and in a mechanical shaker (200 rpm) in triplicate. To investigate the effect of solution pH on the adsorption of Pb onto HCC-Mg, 0.5 g L^−1^ of HCC-Mg was agitated with 25 mL of Pb^2+^ solution (200 mg/L) for 120 min at different pH values (ranging from 2.0 to 5.0). For the kinetic study, 1 g/L of HCC-Mg was mixed with 200 mL of a 200 mg/L Pb (II) solution at pH 5.0. The suspension was shaken for different intervals (ranging from 2 to 120 min). After the contact, the mixture was filtered, and the remaining Pb concentration and the released Mg concentration in the solution were measured using atomic absorption spectrometry (AAS, Perkin-Elmer, AAnalyst 900 T, Waltham, MA, USA). The obtained data were analyzed using the pseudo-first-order, pseudo-second-order, and intraparticle diffusion kinetic models [22,23,24] (Sp 1). For the isothermal study, 1 g/L of HCC-Mg was mixed with Pb solutions of varying concentrations (10 to 300 mg/L). The suspensions were shaken for 120 min, filtered, and AAS was used to measure the equilibrium Pb concentration. The data were analyzed using the Langmuir, Freundlich, Redlich–Peterson, Dubinin–Radushkevich, and Sips isotherm models [25,26,27,28,29,30,31] (Sp 2).

The results were processed and analyzed using Origin 9 software, while data visualization and statistical analysis were performed with the aid of Python 3.13.3 and Matplotlib 3.10.3.

### 2.5. Recycling Experiment

An adsorption–desorption study was conducted over five cycles to examine the possibility of regeneration of HCC-Mg. A 1 g/L Pb-loaded HCC-Mg sample was mixed with 0.1 M HNO_3_ and stirred on a mechanical shaker for 120 min at 200 rpm and room temperature. After the reaction, the suspension was filtered, and the remaining Pb concentration in the solution was measured using AAS. After filtration, the regenerated HCC-Mg was reused for a new cycle of adsorption–desorption experiments.

## 3. Results and Discussion

### 3.1. Characterization

**Figure 1:**
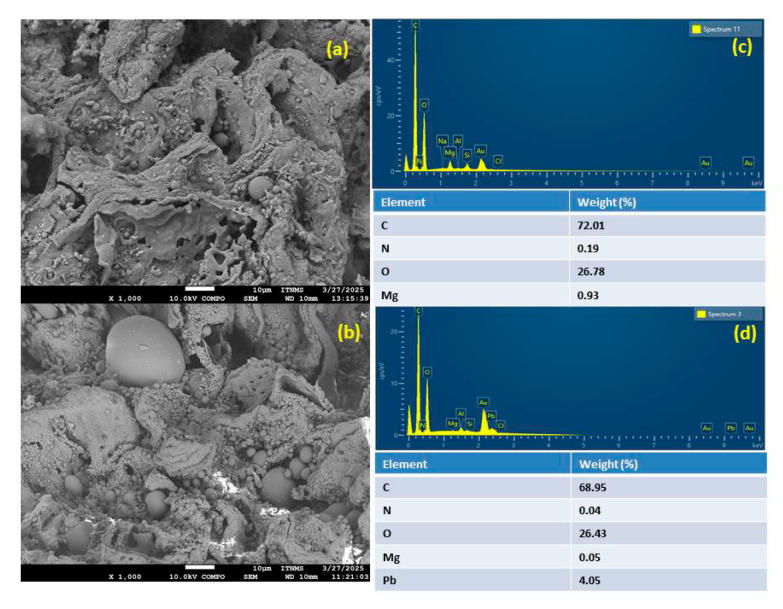
SEM micrographs of HCC-Mg (**a**) before and (**b**) after Pb adsorption and EDX spectra of HCC-Mg (**c**) before and (**d**) after Pb adsorption.

The SEM micrographs of HCC-Mg before and after Pb adsorption are shown in Figure 1. As observed in Figure 1a, the synthesized HCC-Mg material exhibited a highly porous structure prior to Pb adsorption, while after adsorption (Figure 1b), the pores appeared less distinct and partially filled, indicating that Pb ions had occupied the porous surface of the material. Moreover, no Pb crystals were detected on the surface of HCC-Mg, indicating that lead had been adsorbed onto the surface rather than precipitating.

To confirm the presence of Pb on the surface of the HCC-Mg, EDX spectra before and after metal adsorption were investigated (Figure 1c,d). As can be seen from the spectra, upon the adsorption, the Pb peak became noticeable at a content of 4.05% (Figure 1d). In addition, the significant reduction in the Mg peak intensity and the decrease in weight could suggest ion-exchange interaction between Mg and Pb ions (as further confirmed in this study).

**Figure 2:**
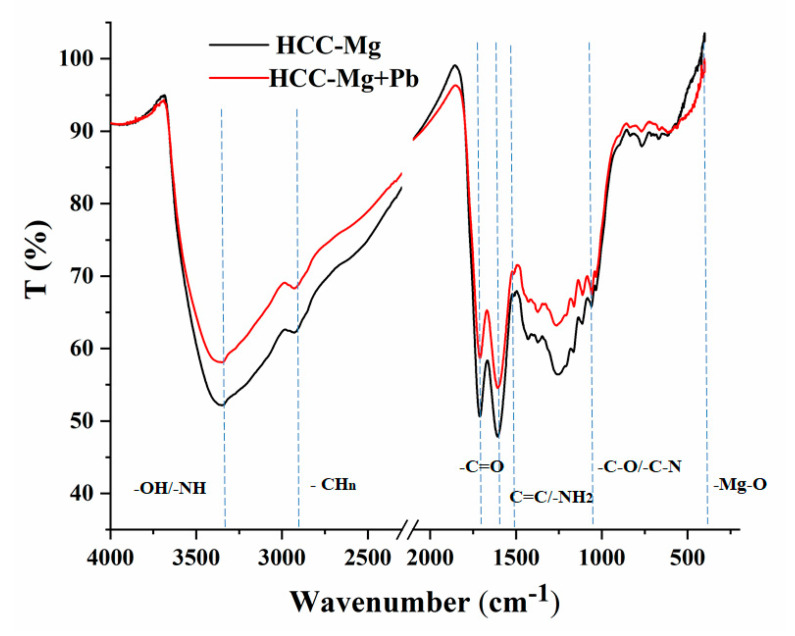
The FTIR of HCC-Mg before and after Pb adsorption.

The surface functional groups of HCC-Mg and the changes caused by Pb adsorption were analyzed using FTIR analysis. Table 1 provides a tabular representation of the observed peaks, their corresponding wavelengths, and their assignments. The FTIR spectra of HCC-Mg (Figure 2) exhibited characteristic peaks that suggested an aromatic structure enriched with oxygen-containing functional groups, a typical feature of hydrochars derived from lignocellulosic precursors [32]. In addition, the FTIR spectra of all HCC-Mg displayed peaks near 450 cm^−1^, which can be attributed to Mg-O bonds, suggesting the successful integration of Mg ions into the HCC structure during the modification process [32,33].

Adsorption of Pb ions over HCC-Mg led to visible differences in the FTIR spectra (Figure 2). Following the adsorption of the metal, all peak intensities were reduced, with some peaks shifting to lower or higher wave numbers. These included the broad peaks centered at 3350 cm^−1^ (–OH, –NH) and 2930 cm^−1^ (–CH_n_), which were reduced in intensity. This decrease suggests a potential complexation process between Pb and HCC-Mg [20]. The pronounced peaks at around 1710 cm^−1^ and 1610 cm^−1^ assigned to C=O from HCC-Mg also weakened, indicating a Pb^2+^–π bond between Pb and the COO^-^ groups on the HCC-Mg surface. Also, the shift to the higher wave number of the adsorption peak situated at 1526 cm^−1^ is indicative of the availability of –NH_2_ groups, which can interact with Pb ions. The participation of –C-O and –C-N groups in the complexation of metal ions was further confirmed with the shift to a higher wave number of the adsorption peak at 1033 cm^−1^ [21]. Considering these variations in FTIR spectra, it can be concluded that –OH, –COOH, and –NH_2_ were the main functional groups responsible for the adsorption of Pb ions on HCC-Mg.

### 3.2. Impact of the pH on Pb Adsorption

The effect of pH on the adsorption of Pb onto HCC-Mg was examined within the pH range of 2 to 6, since Pb tends to precipitate at higher pH values, which could interfere with the process. As shown in Figure S1, the amount of Pb adsorbed onto HCC-Mg increased with rising pH. This trend can be attributed to the decrease in the concentration of hydrogen ions (H^+^) with increasing pH, which reduced the competition between H^+^ and Pb^2+^ ions for active adsorption sites on the material surface. Additionally, the increase in pH facilitated the deprotonation of surface functional groups (such as –COOH and –OH), enhancing the surface negative charge density. The increased negative charge strengthened the electrostatic attraction between the HCC-Mg surface and the positively charged Pb^2+^ ions, ultimately promoting more efficient adsorption [38].

**Figure 3:**
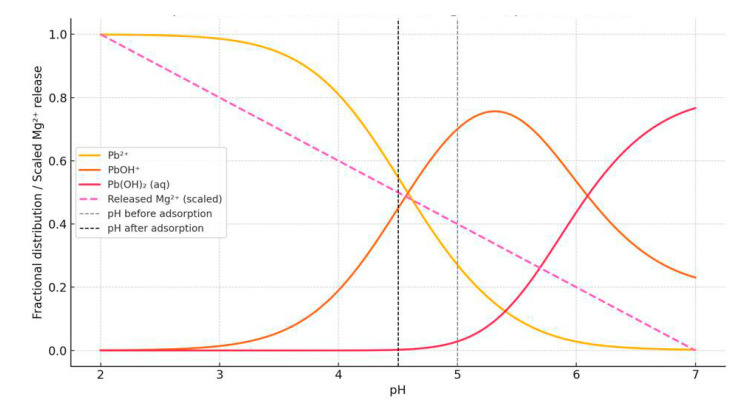
Speciation of Pb^2+^ and release of Mg^2+^ in aqueous solutions.

Supporting this observation, the speciation diagram (Figure 3) shows that Pb^2+^ remained the dominant species at pH values between 4.5 and 5.0, confirming its availability for adsorption in this pH range. Simultaneously, a notable increase in Mg^2+^ concentration (from 1.67 to 3.23 mg/L) indicates ion exchange as a contributing mechanism, where Pb^2+^ ions replaced Mg^2+^ on the adsorbent surface. Additionally, the observed pH drop during the process further suggests the release of protons from surface groups, supporting a combined adsorption mechanism involving both ion exchange and electrostatic interactions.

### 3.3. Kinetic Study

**Figure 4:**
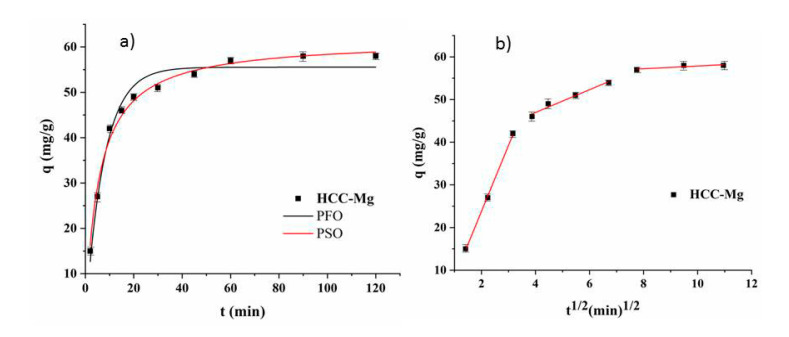
PFO, PSO (**a**), and intraparticle diffusion (**b**) models of Pb adsorption onto HCC-Mg (1 g/L; C_i_ 200 mg/L; pH 5.0).

The experimental data related to the kinetic study revealed rapid removal during the initial 40 min, followed by a slower adsorption process. Equilibrium was achieved after 60 min, with a q_e_ of 60.07 mg/g HCC-Mg when initial Pb concentration was 200 mg/L (Figure 4).

The pseudo-first-order (PFO), pseudo-second-order (PSO), and intraparticle diffusion models were used to describe the kinetics of Pb onto HCC-Mg. The calculated kinetic parameters are presented in Table 2. According to non-linearized PFO and PSO equations (Figure 4a), the adsorption process followed the PSO model better, with R^2^ = 0.991. This indicates that the chemisorption mechanism was involved in the interaction between Pb and HCC-Mg [39,40], which was previously confirmed by FTIR analysis in this study. Additionally, the intraparticle diffusion curve (Figure 4b) shoved multilinearity, indicating that more than one step was involved in the Pb adsorption process onto HCC-Mg. In the first step, Pb ions diffused through a solution to the HCC-Mg surface. During the second step, Pb diffused through the HCC-Mg surface, occupying the adsorption sites, and the equilibrium was reached in the third step [41,42].

### 3.4. Isotherm Study

**Figure 5:**
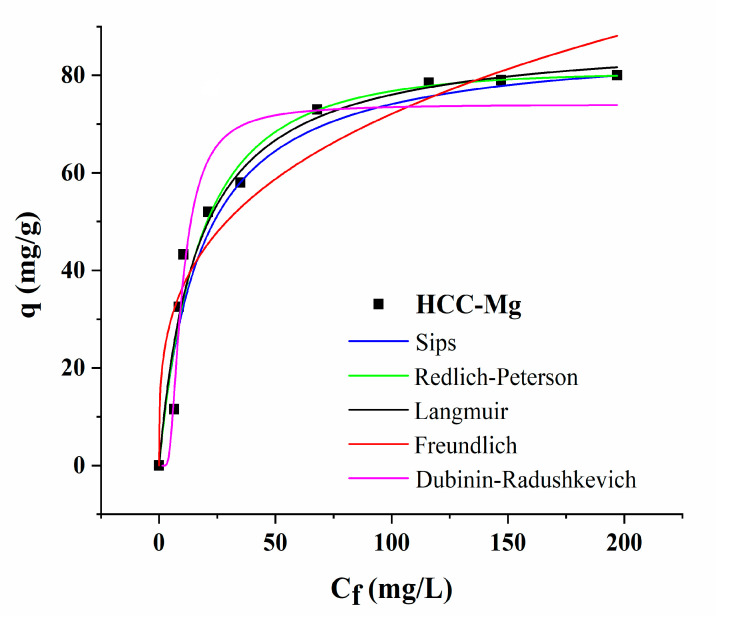
Isotherm models of Pb adsorption onto HCC-Mg (1 g/L; 120 min; pH 5.0).

Two-parameter (Langmuir, Freundlich, and Dubinin–Radushkevich) and three-parameter isotherm models (Sips and Redlich–Peterson) were applied to evaluate which model best described the adsorption process (Figure 5). Based on tabular parameters (Table 3), the Sips model fitted Pb adsorption onto HCC-Mg better, with R^2^ = 0.997. Since the Sips model is a combination of the Freundlich and Langmuir models, the agreement with this model indicates that Pb adsorption onto HCC-Mg occurred as a heterogeneous process involving multiple mechanisms. The R_L_ value between 0 and 1 (Langmuir constant) and n_F_ value between 0 and 10 (Freundlich model) indicates favorable adsorption of Pb onto HCC-Mg [42]. In addition, the Dubinin–Radushkevich (D–R) isotherm model was also considered in this study to provide insight into the nature of the adsorption process. The low value of E (less than 8 kJ/mol) suggests that the adsorption process was predominantly physical (physisorption), as it indicated weak van der Waals interactions rather than strong chemical bonding. This is further supported by the q_DR_ value, which represents the maximum amount of Pb that can be adsorbed onto the HCC-Mg surface, aligning with the results from other models. The K_DR_ constant, which reflects the heterogeneity of the adsorbent surface, indicates a surface with varied adsorption sites and different affinities for Pb ions. The low adsorption energy obtained from the D–R model suggests that the overall process was predominantly physical in nature, involving weak interactions. However, FTIR results provided evidence of specific interactions such as complexation and π–π interactions between Pb ions and surface functional groups, indicating that Pb adsorption onto HCC-Mg proceeded through multiple mechanisms on a heterogeneous surface. These findings suggest that while the adsorption was energetically mild (as indicated by the D–R model), it involved specific surface interactions beyond simple physisorption.

Significantly, the maximum adsorption capacity determined by the Sips model indicated that the HCC-Mg could adsorb 87.08 mg of Pb/g of HCC-Mg (Table 3). When comparing these results to raw CC and other materials reported in the literature, it is evident that the modified HCC-Mg exhibited a significantly better performance than the unmodified CC, demonstrating a 14-fold increase in capacity (Table 4). This notable enhancement in adsorption capacity can be attributed to the modification and conversion of CC into HCC-Mg, which introduced additional functional groups such as –OH, –COOH, –NH_2_, and Mg. These modifications resulted in a much higher number of active sites, significantly boosting the material’s efficiency in adsorbing Pb ions, compared with the untreated CC.

While the two-step HTC and pyrolysis process led to a significant 14-fold improvement in adsorption capacity compared with raw CC, it is important to acknowledge that this synthesis process does involve some additional costs. However, the use of CC, an abundant agricultural waste product available in large quantities (measured in metric tons) after harvest, offers a major advantage in terms of raw material availability. The conversion of CC into HCC-Mg not only enhances Pb adsorption properties but also makes the handling of this waste material easier and more efficient, contributing to the material’s long-term sustainability. By transforming waste into a valuable hydro-pyrochar, this process helps extend the useful life of the raw material. Despite the higher synthesis costs, the modified HCC-Mg material’s exceptional efficiency in removing Pb ions justifies its use, especially in applications where heavy metal removal is a key concern. Given the large-scale availability of CC, this approach offers a sustainable solution for water purification, with the added benefit of being reusable over multiple cycles.

### 3.5. Desorption Study

**Figure 6:**
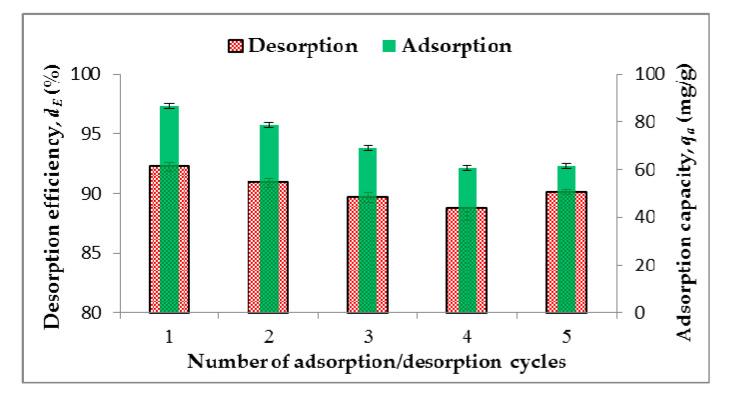
Reusability of HCC-Mg.

The possibility of reusability should be tested to demonstrate the potential of using HCC-Mg in practical applications. The synthesized material between each Pb adsorption cycle was regenerated with 0.1 M HNO_3_ to remove Pb. The adsorption capacity decreased as the number of cycles increased, while the desorption efficiency during all five cycles was above 90% and did not change significantly (Figure 6). A similar trend in the Pb ion removal efficiency was also observed during the Pb-removal tests of NaOH-activated Paulownia leaf hydrochar, sulfide-modified magnetic pinecone-derived hydrochar, dithiocarbonate-modified bamboo hydrochar, and graphene-oxide composite biochar [46,56,57,58]. These results indicate that synthesized HCC-Mg pyro-hydrochar can be efficiently regenerated and reused in further adsorption cycles.

### 3.6. Proposed Adsorption Mechanism

The point of zero charge (pHpzc) of the material was carefully determined (Figure S2), yielding a value of 6.50. The pHpzc is a crucial parameter that indicates the pH at which the surface of the material has no net charge, resulting in neutral interactions with surrounding ions. In this study, Pb ion removal was conducted at pH 5.0, which is lower than the pHpzc of HCC-Mg. At this pH, the surface of the material becomes predominantly positively charged, which would typically cause electrostatic repulsion of Pb^2+^ ions (since they are also positively charged). However, despite the expected repulsion, significant Pb^2+^ removal still occurred, suggesting that other adsorption mechanisms were strongly involved.

**Figure 7:**
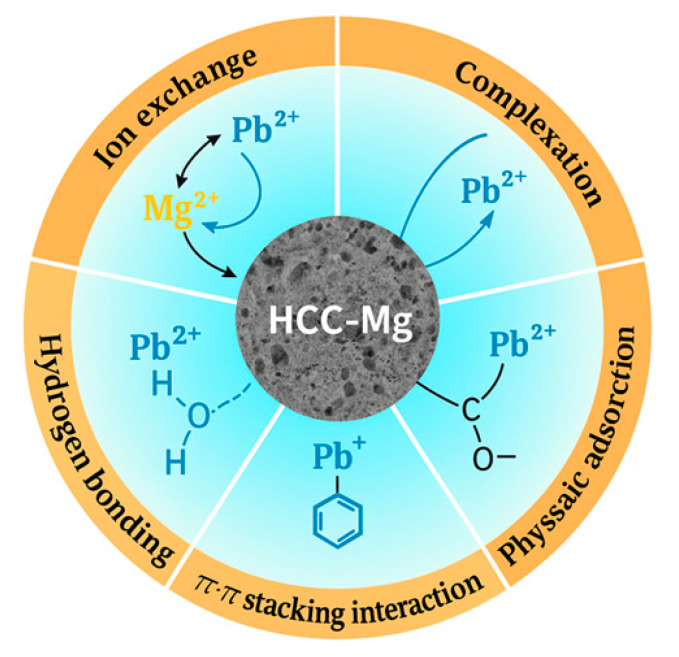
Proposed adsorption mechanisms involved in the removal of Pb ions onto HCC-Mg.

These mechanisms include ion exchange, surface complexation, and specific chemical interactions (Figure 7). The ion exchange process was facilitated by the surface modification, which increased the Mg^2+^ content, enhancing the number of available exchange sites for Pb^2+^. This increased surface Mg^2+^ played a critical role in the overall adsorption process by promoting ion exchange with Pb^2+^.

In addition to ion exchange, surface complexation between Pb^2+^ ions and the oxygen-containing functional groups (e.g., –COOH, –OH) on the HCC-Mg surface contributed to Pb removal. The FTIR analysis revealed that these functional groups were indeed present and may have interacted with Pb^2+^ to form stable complexes. The presence of these oxygenated groups further strengthened the adsorption, even in the presence of weaker electrostatic interactions.

Additionally, hydrogen bonding and π–cation interactions may have also played a role. Pb^2+^ ions can interact with the oxygen atoms in the surface functional groups, and with π-electron-rich sites from aromatic structures within the lignocellulosic content of the material. The interaction between Pb^2+^ and the delocalized π electrons in these structures contributed to the overall stabilization of the adsorbed Pb ions.

In terms of the π–π stacking interaction, this mechanism specifically involves the π orbitals from aromatic rings or conjugated structures (such as those present in lignocellulosic components of HCC-Mg). These π orbitals are delocalized electron clouds associated with the double bonds of the aromatic rings. When two aromatic rings approach each other, their π orbitals can overlap, leading to an interaction known as π–π stacking. This interaction is typically stabilizing and is highly dependent on the relative orientation of the aromatic rings (face-to-face or edge-to-face).

The π–π binding interaction occurs between the π orbitals of adjacent aromatic rings, and this overlap forms a stabilizing interaction due to the electron density between the rings. In addition, π–π interaction may involve π orbitals from conjugated systems donating electron density into vacant orbitals of Pb^2+^ ions (such as the six p orbitals), further contributing to the stabilization of Pb^2+^ on the surface.

The synergistic combination of these mechanisms—ion exchange, surface complexation, hydrogen bonding, π–cation interactions, and π–π binding interactions—explains the high efficiency of Pb^2+^ removal by HCC-Mg. While electrostatic interactions are likely weak due to the positively charged surface at pH 5.0, the diverse functional groups and the enhanced ion-exchange capacity due to Mg functionalization ensure effective adsorption. Thus, Pb^2+^ adsorption onto HCC-Mg is a multi-mechanistic process dominated by chemical binding, ion exchange, and π–π interactions, rather than solely by electrostatic attraction.

## 4. Conclusions

Using a simple two-step approach comprising hydrothermal carbonization followed by pyrolysis in a magnesium-rich environment, the present study demonstrated that the adsorptive performance of corn cob for Pb ions can be significantly enhanced. This process yielded a novel adsorbent material (HCC-Mg) with an adsorption capacity more than 14 times higher than that of raw corn cob (CC), confirming the effectiveness of this straightforward and sustainable modification strategy. In addition to improving Pb adsorption, the conversion of CC, a widely available agricultural waste product, contributes to waste valorization and resource sustainability by transforming biomass into a value-added material.

The kinetic study revealed that chemisorption plays a significant role in Pb adsorption, with intraparticle diffusion exerting a dominant influence on the adsorption kinetics. Isotherm analysis indicated that Pb adsorption onto HCC-Mg was favourable, occurred as a heterogeneous process, and achieved a maximum adsorption capacity of 87.08 mg/g. The adsorption mechanism was found to involve multiple concurrent processes, including ion exchange, surface complexation, Pb–π interactions, and hydrogen bonding, highlighting the complex physicochemical interactions responsible for high removal efficiency.

Given its high adsorption performance, low cost, and environmentally friendly synthesis, and the abundance of raw material, HCC-Mg exhibits great potential for practical large-scale applications in wastewater treatment and the remediation of Pb-contaminated environments. Future research should focus on evaluating the performance of HCC-Mg under real wastewater conditions, studying the effects of co-existing ions and temperature, and further optimizing the material properties to enhance selectivity and reusability.

## Figures and Tables

**Table 1:** Characteristic functional groups in HCC-Mg.

Peak Position (cm^−1^)	Assigments	References
3350	–OH/–NH	[34]
2930	–CH_n_	[35]
Near 1710	C=O	[36]
Near 1610	C=O	[37]
Near 1526	C=C/–NH_2_	[34,37]
1033	–C-O/–C-N	[36]
450	–Mg-O	[32,33]

**Table 2:** Kinetic parameters of Pb adsorption onto HCC-Mg.

*q_e,exp_*	mg/g	60.07			
**PFO**			**Intraparticle Diffusion**		
*q_e,cal_*	mg/g	55.54	*k_i1_*	mg g/min^1/2^	15.46
*k_1_*	1/min	0.13	*R^2^*		0.999
*R^2^*		0.972	*k_i2_*	mg g/min^1/2^	2.66
**PSE**			*R^2^*		0.969
*q_e,cal_*	mg/g	61.63	*k_i3_*	mg g/min^1/2^	0.32
*k_2_*	g/mg min	0.0029	*R^2^*		0.791
*R^2^*		0.991			

**Table 3:** Isotherm parameters of Pb adsorption onto HCC-Mg.

Two-Parameter Isotherm Models		Three-Parameter Isotherm Models	
**Langmuir**		**Sips**	
*q_max_* (mg g^−1^)	88.43	*q_max_* (mg g^−1^)	87.08
*K_L_* (L mg^−1^)	0.061	*K_S_* (mg L^−1^)^−1/ns^	0.057
*R^2^*	0.995	*n_S_*	1.01
		*R^2^*	0.997
**Freundlich**		**Redlich–Peterson**	
*K_F_* (mg g^−1^ (mg L^−1^)^−*1/nf*^)	18.44	*K_RP_* (L g^−1^)	4.764
*n_F_*	3.38	*a_RP_* (mg L^−1^)^-g^	0.037
*R^2^*	0.907	*g*	1.068
		*R^2^*	0.963
**Dubinin–Radushkevich**			
*q_DR_* (mg g^−1^)	74.05		
*K_DR_* (mol^2^ J^−1^)	67.76		
*E* (KJ mol^−1^)	0.086		
*R^2^*	0.949		

**Table 4:** Adsorption capacities of different materials.

Used Material	Preparation Method	pH	Temp (°C)	q (mg/g)	Reference
Corn cob, raw	None	5.0	25	5.95	[19]
Sugarcane bagasse biochar	Pyrolysis	5.0	25	21.0	[43]
Luffa peels	HNO_3_/NaOH modification	5.5	25	34.0	[44]
Corn straw biochar/rGO composite	HTC	5.5	30	34.02	[45]
Sludge-derived biochar	Pyrolysis	5.0	25	30.88	[46]
Prosopis africana shell biochar	Pyrolysis + HTC	5.0	25	45.3	[47]
Chamomile flowers	HNO_3_/NaOH modification	5.0	25	49.5	[44]
Paulownia leaf hydrochar	HTC	5.0	25	49.62	[48]
Pine wood hydrochar	Oxone-modified hydrochar	5.0	25	46.7	[49]
Corn stalk biochar	pyrolysis	5.0	25	20.8	[50]
Waste activated sludge biochar	Anaerobic digestion + pyrolysis	5.0	25	53.96	[51]
Peanut hull biochar	Pyrolysis	5.5	25	63.09	[52]
Modified chicken feather hydrochar	Mg-modified hydrochar	5.0	25	70.41	[53]
Peanut shell biochar	Pyrolysis	5.0	25	82.5	[54]
Orange peel biochar	Pyrolysis	5.0	25	86.96	[55]
HCC-Mg (this study)	HTC + MgCl_2_ pyrolysis	5.0	25	87.05	This study

## Data Availability

The original contributions presented in this study are included in the article/Supplementary Materials. Further inquiries can be directed to the corresponding author.

## Supplementary Materials

The following supporting information can be downloaded at: https://www.mdpi.com/article/10.3390/toxics13060459/s1, Sp 1: Kinetic study; Sp 2: Isotherm study; Figure S1: The effect of initial pH on the Pb adsorption; Figure S2: Ratio of the initial (pH_i_) and final (pH_f_) pH values of sorbents.
