# Supplementary material for: Efficient Adsorption of Lead on Hydro-Pyrochar Synthesized by Two-Step Conversion of Corn Cob in Magnesium Chloride Medium
Source: Toxics. 2025 May 30;13(6):459. doi: 10.3390/toxics13060459 (PMC12196895; doi:10.3390/toxics13060459)
Supplement: Supplementary file 1 [file toxics-13-00459-s001.zip › toxics-3610450-Supplementary Data.pdf]

Supplementary Data

Efficient adsorption of lead on hydro-pyrochar synthesied by two-step conversion of corn cob in magnesium chloride medium

Marija Simić[1], Jelena Petrović[1], Marija Koprivica[1], Marija Ercegović[1], Jelena Dimitrijević[1], Nikola Vuković[1], Núria Fiol Santalo[2]

[1] Institute for Technology of Nuclear and Other Mineral Raw Materials, Belgrade, Serbia

[2] LEPAMAP-PRODIS Research Group, Universitat de Girona, Spain

Sp1 Kinetic study

The PFE, PSE model and the intraparticle diffusion model can be expressed as Equations (1)–(3), respectively [22,23,24]:

$$1/q_t=1/q_e+k_1/q_e t \quad (1)$$

$$t/q_t=1/k_2 q^2_e+t/q_e \quad (2)$$

$$q_t=k_{id} t^{1/2}+C \quad (3)$$

where: k_1 and k_2 are the pseudo-I-order and pseudo-II-order rate constant (min^{-1}), respectively; k_{id} is the intraparticle diffusion rate constant (mg g^{-1} $min^{-1/2}$), and C is intercept.

Sp 2 Isotherm study

The Langmuir isotherm model is valid for monolayer adsorption where the sorption of species can occur only at a fixed number of energetically equivalent sites. This model was used in order to get information about the maximum biosorption capacity of the biosorbent. The Langmuir isotherm model can be expressed as the following equation [25]:

$$q_e=q_{max} K_L C_e/(1+K_L C_e) \quad (4)$$

where: q_{max} is the maximal amount of adsorbed Pb on the HCC-Mg (mg g^{-1}), and K_L is the Langmuir constant (L mg^{-1}).

The dimensionless constant (R_L) which describes the feasibility of the Langmuir isotherm model and favorability of the biosorption process can be expressed by the following equation [26]:

$$R_L=1/(1+K_LC_0) \quad (5)$$

where: R_L is separation factor. The biosorption process of Pb on the HCC-Mg is: unfavorable if the $R_L > 1$; linear if the $R_L = 1$; favorable if the $0 < R_L < 1$ or irreversible if the $R_L = 0$ [27].

The Freundlich isotherm model is valid for multilayer adsorption where the sorption process occurs at the heterogeneous surface. This model can be expressed as the following equation [28]:

$$q_e=K_FC^{1/\mathrm{nF}} \quad (6)$$

where: K_F is the Freundlich constant (mg g^{-1} (mg $L^{-1})^{-1/n}$) and $1/n_F$ is an empirical Freundlich parameter. The biosorption process of Pb on the HCC-Mg is favorable if the value of n_F is between 1 and 10.

The Dubinin–Radushkevich isotherm model was used for the calculation of sorption energy. It can be expressed as the following equations [29]:

$$q_e=q_{\mathrm{DR}}\mathrm{e}^{-K_{DR}\varepsilon^2} \quad (7)$$

$$\varepsilon=RTln[1+1/Ce] \quad (8)$$

where: q_{DR} is the theoretical isotherm saturation capacity (mg g^{-1}); K_{DR} is the Dubinin–Radushkevich constant (mol^2 kJ^{-2}); ε is the Polanyi potential (KJ^2 mol^{-2}); R is the gas constant (8.314 J (mol $K)^{-1}$); and T is the absolute temperature (K).

The Sips isotherm model is the combination of the Langmuir and Freundlich isotherm models: it approaches the Freundlich isotherm at low sorbate concentrations, while at high

concentrations it predicts a monolayer adsorption capacity characteristic for the Langmuir isotherm. It can be expressed as the following equation [30]:

$q_e = q_{max}(K_S C_e)^{1/ns}/(1+(K_S Ce)^{1/nS})$ (9)

where: K_S is the Sips constant related to sorption affinity ((mg L^{-1})$^{-1/n}$) and 1/n_S is an empirical Sips parameter which indicates sorbent heterogeneity; a higher value of this parameter indicates a more heterogenous system.

The Redlich–Peterson isotherm model can be expressed as the following equation [31]:

$q_e = K_{RP} C_e/(1+a_{RP} C_g e)$ (10)

where: K_{RD} and a_{RP} are Redlich–Peterson constants (L g^{-1}) and ((mg L^{-1})$^{-g}$), respectively, and g is an empirical Redlich–Peterson parameter ($g \leq 1$).

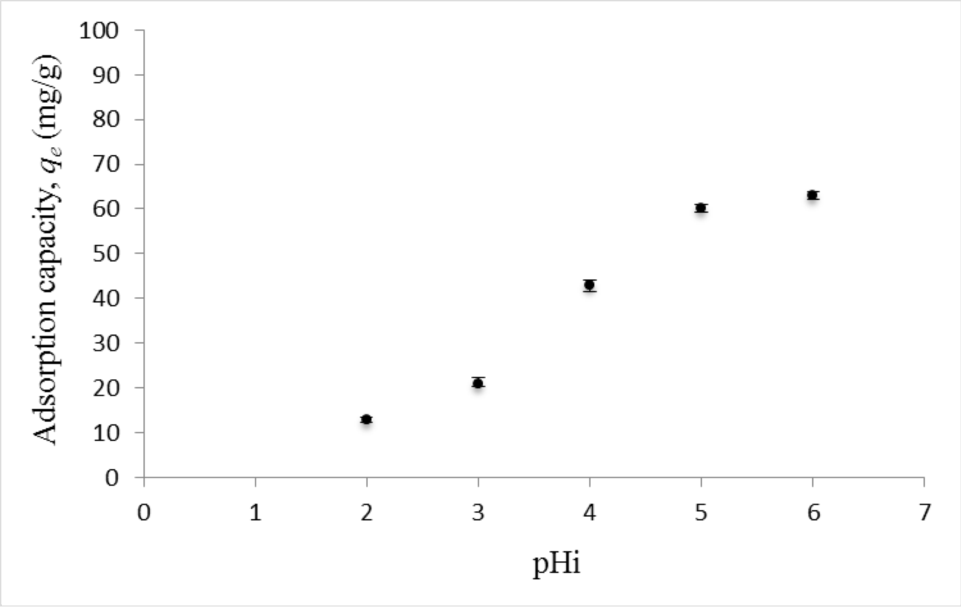

Fig. S1. The effect of initial pH on the Pb adsorption

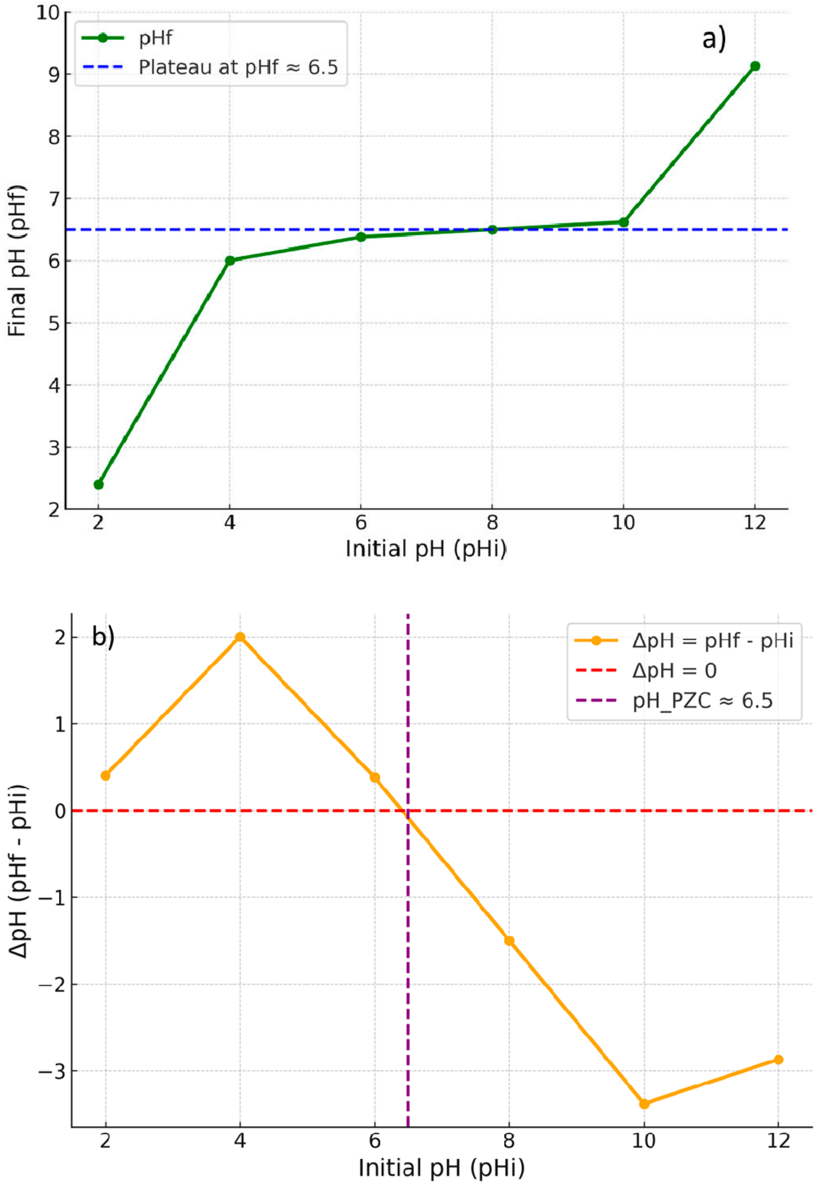

Fig. S2. Ratio of the initial (pH_i) and final (pH_f) pH values of sorbents (a), and ΔpH vs. pH initial (b).
